# Isolate-Based Surveillance of *Listeria monocytogenes* by Whole Genome Sequencing in Austria

**DOI:** 10.3389/fmicb.2019.02282

**Published:** 2019-10-01

**Authors:** Adriana Cabal, Ariane Pietzka, Steliana Huhulescu, Franz Allerberger, Werner Ruppitsch, Daniela Schmid

**Affiliations:** ^1^Austrian National Reference Centre for Listeria, Austrian Agency for Health and Food Safety, Vienna, Austria; ^2^European Public Health Microbiology Training Programme (EUPHEM), European Centre for Disease Prevention and Control (ECDC), Stockholm, Sweden

**Keywords:** *Listeria monocytogenes*, whole-genome sequencing, cgMLST, food isolates, cluster, surveillance, trace back

## Abstract

*Listeria monocytogenes* (*L. monocytogenes*) is a ubiquitous organism that can easily enter the food chain. Infection with *L. monocytogenes* can cause invasive listeriosis. Since 2014, in Austria, *L. monocytogenes* isolates from human and food/food-associated samples have been provided on a mandatory basis by food producers and laboratories to the National Reference Laboratory. Since 2017, isolates undergo routinely whole genome sequencing (WGS) and core genome Multilocus Sequence Typing (cgMLST) for cluster analyses. Aims of this study were to characterize isolates and clusters of 2017 by using WGS data and to assess the usefulness of this isolate-based surveillance for generating hypotheses on sources of invasive listeriosis in real-time. WGS data from 31 human and 1744 non-human isolates originating from 2017, were eligible for the study. A cgMLST-cluster was defined as two or more isolates differing by ≤10 alleles. We extracted the sequence types (STs) from the WGS data and analyzed the food subcategories meat, fish, vegetable and diary for associations with the ten most prevalent STs among food, through calculating prevalence ratios (PR) with 95% confidence intervals (CI). The three most frequent STs among the human isolates were ST1 (7/31; 22.6%), ST155 (4/31; 12.9%) and ST451 (3/31; 9.7%) and among the non-human isolates ST451 (614/1744; 35.2%), ST8 (173/1744, 10.0%) and ST9 (117/1744; 6.7%). We found ST21 associated with vegetables (PR: 11.39, 95% CI: 8.32–15.59), ST121 and ST155 with fish (PR: 7.05, 95% CI: 4.88–10.17, PR: 3.29, 95% CI: 1.86–5.82), and ST511, ST7 and ST451 with dairy products (PR: 8.55, 95% CI: 6.65–10.99; PR: 5.05, 95% CI: 3.83–6.66, PR: 3.03, 95% CI: 2.02–4.55). We identified 132 cgMLST-clusters. Six clusters contained human isolates (ST155, ST1, ST101, ST177, ST37 and ST7) and for five of those cgMLST-based cluster analyses solely was able to hypothesize the source: an Austrian meat processing company, two Austrian cheese manufacturers and two vegetable processing companies, one based in Austria and the other in Belgium. Determining routinely STs in food isolates by WGS allows to associate STs with food products. Real-time WGS of *L. monocytogenes* isolates provided mandatorily, proved to be useful in promptly generating hypotheses on sources of invasive listeriosis.

## Introduction

*Listeria monocytogenes* (*L. monocytogenes*) is a Gram positive bacterium, able to survive in extreme environmental conditions, which enables the microorganism to cause food-borne infections and outbreaks (Buchanan et al., 2017). Invasive listeriosis mainly affects immunocompromised individuals, pregnant women and the elderly (de Noordhout et al., 2014; Buchanan et al., 2017). Contamination with *L. monocytogenes* can occur at any stage of the food chain (farm, production and retail). Sliced products, in particularly meat, have a significantly higher risk of being contaminated with *L. monocytogenes* compared to unsliced products (Chaitiemwong et al., 2014; Gómez et al., 2015).

Rapid and accurate typing methods of *L. monocytogenes* isolates are indispensable for timely identification of molecular clusters of isolates originating from food products, food-associated surfaces and patients with listeriosis (Radoshevich and Cossart, 2017). Whole Genome Sequence (WGS)-based typing, and especially core genome (cg)Multilocus Sequence-based Typing, is nowadays the preferred method for characterization of *L. monocytogenes* by genoserogroup (SG), clonal complex (CC), sequence type (ST), complex type (CT) and for cluster analyses to hypothesize the sources of invasive listeriosis, in particular, when information on patients’ food exposure is lacking (Buchanan et al., 2017; Moura et al., 2017; Lüth et al., 2018). In Austria, in the year 2017, WGS replaced PFGE for typing and cluster analyses, using the cgMLST scheme established by Ruppitsch and coworkers (Ruppitsch et al., 2015).

Since 2014 it has been mandatory for all Austrian microbiological laboratories (public and private) or food producers to provide *L. monocytogenes* isolates from human cases and food or food-associated environments to the National Reference Laboratory (NRL) for typing (acc. to §38 Abs. 1 Z 6 LMSVG and §74 LMSVG). The non-human isolates are provided with information on the sample origin including food matrix and a laboratory unique identifier. Also in 2014, as a consequence of an outbreak of invasive listeriosis in Austria in 2011 (Schmid et al., 2014), the Federal Ministry of Health classified 600 food-producing companies as being at high risk of contaminating their food production with *L. monocytogenes*, which resulted in an intensified sampling scheme at these companies. These activities together explain an increase in the number of food/food-associated isolates at the NRL (almost doubling between 2014 and 2017). From 2017 onward, any food and food-associated isolate provided to the NRL is routinely whole genome-sequenced. The occurrence of a single case of invasive listeriosis prompts WGS and cgMLST-based cluster analyses with all non-human isolates of the NRL-WGS database. If no cluster with the clinical isolate is found, cgMLST analyses will be extended to food/food-associated isolates, originating from the years 2014 to 2016. In the event of a cluster of the patient isolates with food/food-associated isolates, the national Public Health (PH) authority is informed. At the discretion of the National authority the local authorities are requested to check the food safety measures at the site of the related producers. The occurrence of a phylogenetically related case of listeriosis within 3 months prompts epidemiological investigations including food exposure analysis, tracing analyses and targeted sampling of the incriminated food processor(s) to test the hypothesis on the outbreak source. Clusters containing only non-human isolates are reported quarterly to the national PH authority.

The first aim of this study was to characterize human and non-human isolates from the year 2017 by WGS, describe the cgMLST-clusters found and identify associations between STs and food groups. The second aim was to assess the usefulness of routine WGS of mandatorily provided human and food/food-associated isolates of *L. monocytogenes* for promptly generating and testing hypotheses on sources of invasive listeriosis.

## Materials and Methods

### Isolates of *L. monocytogenes*

WGS data from 1,960 *L. monocytogenes* isolates including 36 human and 1924 non-human isolates, recovered from Austrian samples in the year 2017, were available at the SeqSphere^+^ database (Ridom© GmbH, Münster, Germany). The human isolates originated from patients with invasive listeriosis. The human cases were characterized by month of occurrence and province of residence. The non-human isolates were described by category (i.e., food, food-associated surfaces and environment) and subcategory (food) of origin based on information provided by the referral laboratories.

### Analysis of the WGS Data

From the WGS data, we extracted the SGs, based on the five SG-associated genes, as described (Doumith et al., 2004), the CC (Ragon et al., 2008), the conventional ST, based on the seven housekeeping genes as described by Salcedo et al. (2003), and the CT, based on the previously mentioned cgMLST scheme containing 1,701 target genes (Ruppitsch et al., 2015). In case of a previously unknown ST, a new ST was assigned by the pubMLST database^1^ curators. All isolates from the year 2017 were characterized by SG, CC, ST and CT. We compared the findings on SGs with traditional serotyping by sero-agglutination. For cluster detection, we generated Minimum Spanning Trees (MSTs) based on the core genome targets. A cluster was defined as a group of at least two isolates, human or non-human, differing from each other by ≤10 alleles, according to the previously established cluster threshold (Ruppitsch et al., 2015). An outbreak-cluster was defined as a cluster including human isolates from at least two patients with invasive listeriosis; otherwise we classified the clusters as “single case-cluster” including a clinical isolate from one patient besides non-human isolates or “non-human cluster” including no clinical isolate. The clusters were described by SG, ST and CT. The outbreak cluster and single case-cluster were used to generate hypotheses on the *L. monocytogenes* source by trace-back analyses.

### Statistical Analysis

We analyzed the association between the ten most prevalent *L. monocytogenes* STs and the food subcategories by calculating prevalence ratios (PR) with 95% confidence intervals (CI). Data entry and analyses were performed using STATA 13.0 (STATA Corp., College Station, TX, United States).

## Results

### Study Cases and Isolates

Between January 1 and December 31, 2017, 32 cases of invasive listeriosis (31 culture confirmed) were reported to the listeriosis case-based Austrian surveillance system (incidence 0.36/100,000 inhabitants). Supplementary Figure S1 illustrates the cases by month of occurrence and province of residence. Seven cases peaked in September, including six cases from five neighboring Austrian provinces. Another peak included five cases in December 2017.

After exclusion of isolate duplicates (*n* = 88), isolates of low sequence data quality (≤90% good targets) (*n* = 5), non-invasive isolates (*n* = 2), isolates of *Listeria* species other than *L. monocytogenes* (*n* = 1), non-Austrian isolates (*n* = 49), isolates with no data of origin (*n* = 7) and isolates of external quality assessments (*n* = 33), 1,775 *L. monocytogenes* isolates (31 human and 1744 non-human isolates) remained for WGS-based characterization and cluster analyses. We identified five SGs, 53 CCs, 71 STs and 475 CTs. The findings of the WGS-based genetic profile of the human isolates are displayed in Table 1. Briefly, the 31 human isolates were assigned to SGs IIa, IIb, IIc, IVb and 4a/4c, which was in accordance to the results of the traditional seroagglutination (data not shown). The two most prevalent SGs were IIa (18/31; 58.1%) and IVb (10/31; 32.3%) and the three most prevalent STs were ST1 (7/31; 22.6%), ST155 (4/31; 12.9%) and ST451 (3/31; 9.7%). We found 14 different CCs, 15 STs and 29 CTs.

**TABLE 1:** Human isolates of *L. monocytogenes* (*n* = 31) by SG, CC, ST and CT, recovered from cases of invasive listeriosis in Austria, 2017.

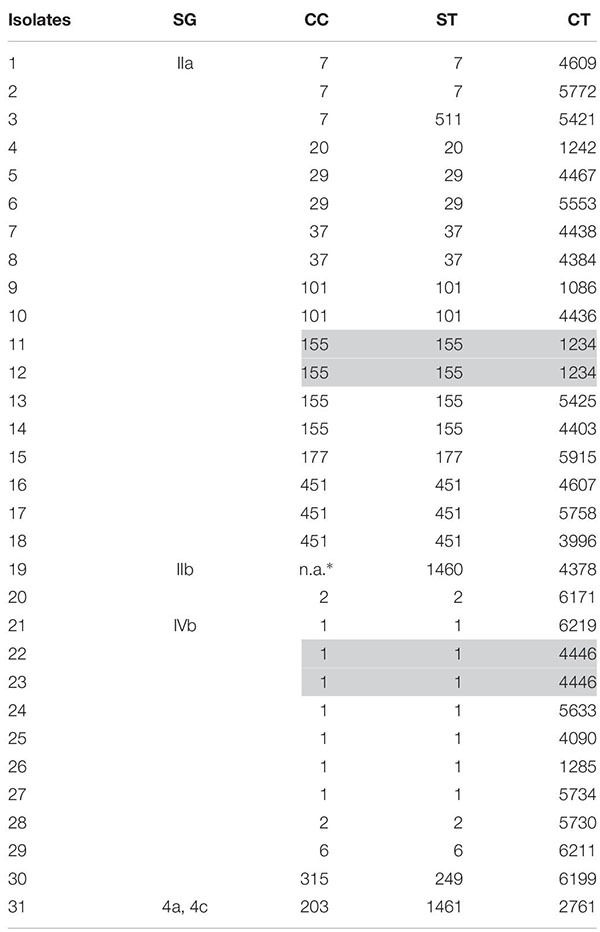				

*^∗^No CC assigned by pubMLST database. Gray rows display isolates from epidemiologically linked cases.*

Among the non-human isolates, 82% belonged to SG IIa (1425/1744), 7.2% to IVb (125/1744), 5.8% to IIc (102/1744), 5.2% to IIb (91/1744) and 0.1% to 4a/4c (1/1744; 0.1%). We identified 44 different CCs, 70 STs and 449 CTs. The three most frequent CCs were CC451 (614/1744; 35.2%), CC8 (173/1744, 10.0%) and CC7 (151/1744; 8.7%) and the three most frequent STs were ST451 (614/1744; 35.2%), ST8 (173/1744, 10.0%) and ST9 (117/1744; 6.7%).

### Isolates by Categories/Subcategories of Sample Origin

Based on the information provided by the referral laboratories or food producers, we could allocate the non-human isolates to three categories of sample origin: “food,” from which 832 isolates originated, “food-associated surface” samples, which yielded 849 isolates, and “others” including mainly environmental isolates (compost, gully, feed and animal samples), from which 63 isolates originated (Table 2). Supplementary Table S1 gives the ten most prevalent STs by category of isolate origin. Among the category food, the ten most frequent STs were 7, 8, 9, 21, 37, 121, 155, 451, 504 and 511, accounting for 70.7% of all food isolates (588/832). The four most frequent subcategories of isolates from food origin were meat (463/832, 55.7%), dairy products (126/832, 14.1%), fish (85/832, 10.2%) and vegetables (75/832, 9%).

**TABLE 2:** Non-human isolates (*N* = 1744) by category of sample origin and food subcategory, Austria, 2017.

**Categories/Sub Categories**	***n***	**%**
**Food**	**832**	**47.7**
Meat	463	
Dairy	126	
Fish	85	
Vegetable	75	
Bread and derivatives	12	
Egg	4	
Mixed food	39	
Seafood	5	
Food not further defined	23	
**Food-associated surface°**	**849**	**48.7**
**Others^∗^**	**63**	**3.6**

*^∗^Animal organs, water, compost, feed and gully. °Secondary product or surface within the food production process.*

### Association Between STs and Food Products

We tested the four most frequent food subcategories of isolate origin, including meat, fish, vegetable and dairy products, for associations with the ten most frequent STs of the food isolates (Table 3). Meat isolates were 1.32 (95%CI: 1.1–1.5), 1.7 (95%CI: 1.6–1.9), 1.31 (95%CI: 1.1–1.5) and 1.66 (95%CI: 1.6–1.8) times more likely to be of ST8, ST9, ST37 and ST504, respectively, compared with the non-meat isolates. Fish isolates were 7.5 (95%CI: 4.9–10.2) and 3.3 (95%CI: 1.9–5.8) more likely to be of ST121 and ST155, respectively, compared with non-fish isolates. Isolates from dairy products were 5.1 (95%CI: 3.8–6.7), 3.03 (95%CI: 2.0–4.6) and 8.6 (95%CI: 6.7–11.0) times more likely to be of ST7, ST451 and ST511, respectively, compared with non-dairy isolates. Vegetable isolates were 11.4 (95%CI: 8.3–15.6) times more likely to be of ST21, compared to non-vegetable products.

**TABLE 3:** The 10 most frequent STs among the 832 food isolates and their association with the four most frequent *L. monocyogenes* positive food subcategories; PR [95%CI], 2017.

**ST**	**Isolates**		**Meat**		**Fish**		**Dairy**		**Vegetable**	
	***n***	**%**	**PR**	**95% CI**	**PR**	**95% CI**	**PR**	**95% CI**	**PR**	**95% CI**
9	110	13.2	1.7	1.6–1.9	0.2	0.0–0.6	0.1	0.0–0.4	0.0	n.a.
121	99	11.9	0.8	0.7–1.0	7.0	4.9–10.2	0.0	n.a.	0.3	0.1–1
511	77	9.3	0.2	0.1–0.4	0.0	n.a.	8.6	6.7–11.0	0.3	0.1–1
8	74	8.9	1.3	1.1–1.5	1.0	0.5–2.0	0.2	0.0–0.7	0.6	0.2–1.6
37	60	7.2	1.3	1.1–1.5	0.0	n.a.	1.1	0.6–2.0	0.2	0.0–1.3
7	42	5	0.4	0.2–0.7	0.0	n.a.	5.1	3.8–6.7	0.6	0.1–2.2
451	41	5	0.5	0.3–0.9	0.5	0.1–2.1	3.0	2.0–4.6	1.3	0.4–3.2
21	30	3.6	0.3	0.1–0.6	0.0	n.a.	0.0	n.a.	11.4	8.3–15.6
504	30	3.6	1.7	1.6–1.8	0.0	n.a.	0.0	n.a.	0.0	n.a.
155	28	3.4	1.1	0.8–1.4	3.3	1.9–5.8	0.0	n.a.	0.0	n.a.

### Description of the Clusters

The cgMLST analyses revealed 303 singletons and 132 clusters containing 1,472 isolates, whereas more than one isolate per sampled food product could have been included in a cluster. Among the 132 clusters, there were two outbreak-clusters (Cluster A and B) and four single case-clusters (Cluster C, D, E and F), as displayed in Table 4, and 126 non-human clusters. Of the 126 non-human clusters, 67 (53.2%) clusters included food isolates only (i.e., food cluster), 12 (9.5%) contained isolates from food-associated surfaces exclusively, five (4.0%) environmental isolates only and other 42 clusters contained a mixture of food, food-associated surface and environmental isolates: 26 food/food-associated surfaces clusters, 12 food/environmental cluster, three food-associated/environmental clusters and one cluster of food/food- associated surface/environmental isolates.

**TABLE 4:** The human isolate including clusters (A–F) described by genetic profile of the causative *L. monocytogenes* strain, by time of case occurrence, and month/year of food/food-associated surface sampling, from which the outbreak/cluster strain originated, Austria, 2015–2018.

**Cluster Type**	**Month/year of case occurrence**	**Month/year of food/food-associated sampling, from which the outbreak/cluster strain originated**	**Food subcategory**	**Description of the clusters**			
				**Cluster ID**	**SG**	**ST**	**CT**
7-case outbreak-cluster	2015, 11 – 2017, 09	2015, 09 – 2018, 05	Meat, fish, mixed food	A	IIa	155	1234
2-case outbreak-cluster	2017, 07, 09	2017, 04 – 2018, 11	Meat	B	IVb	1	4446
Single case-cluster	2017, 09	2017, 03, 12 – 2018, 08	Frozen vegetables	C	IIa	101	4436
Single case-cluster	2017, 11	2017, 11	Frozen vegetables	D	IIa	177	5915
Single case-cluster	2017, 07	2017, 03	Cheese products	E	IIa	37	4438
Single case-cluster	2017, 05	2017, 05	Cheese products	F	IIa	7	4609

When analyzing the ST frequency distribution among the non-human clusters, we found in the 67 food-clusters, ST121 most prevalent (15/67; 22.4%), followed by the ST9 (10/67; 14.9) and ST1 (5/67; 7.5%). Among the other non-human clusters, as described above, there was no predominant ST.

### WGS-Based Surveillance for Generating Hypothesis on Sources of Invasive *Listeriosis*

**FIGURE 1:**
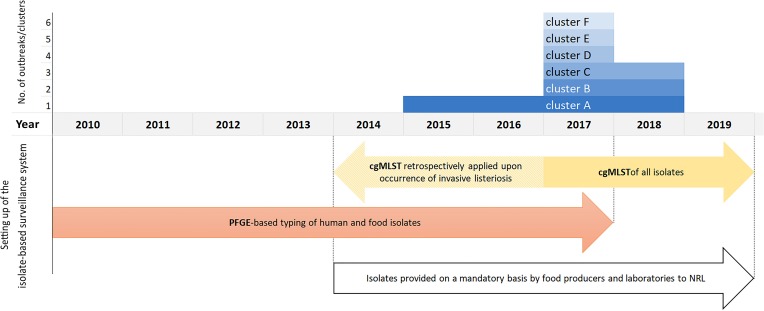
Clusters A–F by the year, when the human and food samples tested positive for the outbreak/cluster strain, and illustration of the setting up of the Austrian isolate-based surveillance of *L. monocytogenes*.

The outbreak-cluster, cluster A (IIa, CC155, ST155, CT1234) contained invasive isolates from seven patients, which occurred from November 2015 to September 2017. The meager information on patients’ food exposure did not reveal any common link. By retrospective cgMLST of food and food-associated surface isolates we were able to identify the outbreak strain among isolates from food/food-associated samples, collected in September and October, 2015. The matching food/food-associated isolates were traced back to an Austrian meat processing company (Pietzka et al., 2019). The outbreak strain was detectable at the company until May 2018. The second outbreak-cluster, cluster B (IVb, CC/ST1, CT4446), contained isolates from two patients, who occurred in July and September 2017. The food isolates of the cluster originated from meat and processed meat samples, which had been collected 3 months prior to the primary outbreak case. The outbreak B strain was detectable in meat samples until November 2018. Each of the four single-case clusters included isolates from one invasive listeriosis case besides food/food-associated isolates: Cluster C (SGIIa, CC/ST101, CT4436), cluster D (SGIIa, CC/ST177, CT5915), cluster E (SGIIa, CC/ST37, CT4438) and cluster F (SGIIa, CC/ST7, CT4609. Cluster C: after the detection of a human case in September 2017, the patient strain was found among isolates, which had been recovered in March 2017, from private samples of an Austrian retailer of frozen vegetable, processed in Belgium. Cluster D: the patient strain matched with isolates from private samples of frozen spinach of a wholesaler of frozen vegetables, collected in the same month as the case occurred. Cluster E was traced back to a small Austrian sheep cheese producer. The cheese isolates originated from a sampling conducted 4 months prior to the case occurrence. For cluster F useful information on the patient food exposure was available to hypothesize the source of infection. Detecting the patient strain in cheese samples, collected from the case-patient’s refrigerator, implicated an Austrian cheese producing company as the most likely source of the invasive listeriosis. Figure 1 illustrates the clusters A-F described by type of cluster (2 outbreak-clusters, 4 single case-clusters), time-span of detection of the outbreak/cluster strain in human and non-human samples and time of implementation of the isolate-based surveillance of *L. monocytogenes*, including the measures adopted to make isolates available for typing as well as the typing methods.

## Discussion

Our study describes for the first time the findings of a WGS-based characterization of human and non-human *L. monocytogenes* isolates and their clusters, detected in the year 2017 in Austria. It also illustrates the usefulness of a mandatory isolate-based surveillance of *L. monocytogenes* combined with routine WGS for promptly generating and testing hypotheses on the sources of invasive listeriosis. Due to the legal obligation for all Austrian microbiological laboratories to provide human and non-human isolates of *L. monocytogenes* to the NRL, the NRL has created a large collection of *L. monocytogenes* isolates from 2014 onward. Since 2017 any provided *L. monocytogenes* isolate has been whole genome-sequenced at the NRL.

ST1 was the most prevalent ST among human isolates of the year 2017. The predominant occurrence of this ST in invasive listeriosis cases has been previously reported (Chenal-Francisque et al., 2011; Huang et al., 2015; European Centre for Disease Prevention and Control [ECDC], 2016; Pontello et al., 2016; Kuch et al., 2018). Almost half of the food isolates with ST1 originated from meat products, despite this ST has been associated to dairy products elsewhere (Maury et al., 2019). ST1 caused a 2-case outbreak in 2017. By using the NRL-WGS database, the outbreak strain could be promptly detected among isolates from meat products, which had been sampled 2 months before the first invasive case occurred. This allowed to generate the hypothesis on the sources of the invasive listeriosis while lacking epidemiological indications. The involved meat products were traced back to five unrelated meat processing companies. The related Ready-To-Eat meat products fulfilled the microbiological safety criteria of the Commission Regulation (EC) No 2073/2005 and the companies were compliant with HACCP concepts. No further case due this strain was reported. The second most common ST among the human isolates, ST155, was the 10th most prevalent one among the food isolates of the year 2017. ST155 was part of two clusters in 2017, a non-human cluster of isolates from fish samples and an outbreak, containing isolates of two cases of invasive listeriosis of the year 2017. A retrospective cgMLST-cluster analysis discovered the outbreak strain among invasive isolates from five patients and among food isolates from samples of the years 2015 and 2016, collected at an Austrian meat processing company. Based on this finding, the company could be suspected as the most likely source of the seven cases-patients, whereas epidemiological information failed to generate a useful hypothesis on the outbreak source. The control measures at the company were evaluated by environmental and food monitoring for *L. monocytogenes* and cgMLST of *L. monocytogenes* isolates, which were still detected in 2017 and the first half of 2018, until the strain was eliminated from the company in May 2018 (Pietzka et al., 2019). ST155 has been previously reported to be related with meat products in Switzerland, but also with fish products in Nigeria (Meier et al., 2017; Nwaiwu et al., 2017). Among the Austrian food isolates from the year 2017, isolates from fish products were three times more likely to be ST155. We found also ST155 associated with meat products, when having included the food isolates from 2018 in the analyses (data not shown). The third most common ST among the human isolates was ST451, causing three cases and accounting for 5% of the food isolates of the year 2017. The three human ST451 isolates and the majority of the food isolates were distinguishable from each other by cgMLST. To our knowledge, ST451 has been found in single patients from Germany and France, in 2017 and 2018 (Moura et al., 2017; Halbedel et al., 2018) and in samples from poultry (Ebner et al., 2015) and a farm environment in Switzerland (Dreyer et al., 2016). None of these strains clustered with the Austrian patient strains of the year 2017. ST451 accounted for 68% of the 2017 food-associated surface isolates, of which 90% were part of a single non-human cluster (CT4422). This could be traced back to a cheese producing company in Austria. Concordantly, we found dairy products three times more likely to be associated with ST451 than other food products. However, ST511 was even stronger associated with dairy products. The two dominating ST511 strains (CT4383 and CT4445) of the 2017 in Austria originated from two different dairy products and were both distinguishable from the one clinical isolate of the year 2017 (15 allelic differences). We found dairy products also strongly related to ST7, which was part of three cgMLST-clusters in 2017. ST37 was identified in 10 non-human cgMLST-clusters, of which nine were associated with meat products. Other studies, carried out in Austria, linked ST37 to non-food processing environments such as compost, soil or water (Linke et al., 2014). A ST37 cluster of the year 2017, containing the isolate from a single case-patient, could be traced back to an Austrian small sheep cheese manufacturer. The matching cheese isolate originated from a sampling, performed 4 months prior to the case occurrence. Again, only the routine WGS of the obligatorily provided isolates to the NRL allowed to promptly hypothesize the most likely source, even for a single case of invasive listeriosis. The same holds true for two cases of invasive listeriosis, one due to *L. monocytogenes* ST101, occurred in September 2017 and the other due to ST177, occurred in December 2017. Without the WGS database from the isolate-based surveillance the sources of both invasive cases could not have been hypothesized as information on food exposure and buying behavior did not give any clue.

*L. monocytogenes* isolates from meat products were found to be associated with the STs 8, 9, and 504. These three STs were part of 23 cgMLST-clusters among meat isolates. As no clinical isolate was involved, there were no further investigations initiated. The associations of ST8 and ST9 with meat products were already described (Henri et al., 2016; De Cesare et al., 2017; Nastasijevic et al., 2017; Fagerlund et al., 2018; Sosnowski et al., 2019). ST121 caused 19 non-human cgMLST clusters in 2017. Association between ST121 and fish products has been previously described (Knudsen et al., 2017; Meier et al., 2017). *L. monocytogenes* isolates from vegetable products showed to be associated with ST21, which was detected in six different non-human clusters among vegetable, meat and food-associated surface isolates, but not in clinical isolates of the year 2017. Interestingly, ST21 was previously reported to be associated with rodents, birds or the environment (Linke et al., 2014; Zuber et al., 2019) but, to our knowledge, is infrequent in humans.

The Austrian isolate-based surveillance of *L. monocytogenes* with routine WGS-based cluster analysis for tracing sources of *L. monocytogenes* contamination could be improved by the following certain measures. In particular, the information given on the origin of the isolates should be ascertained in a standardized way using a uniform questionnaire by the referral laboratories and food producers. Secondly, overestimation of food associations with STs and of cluster extension due to overrepresentation of isolates originated from one sampled food as explained by the sampling procedure (i.e., five subsamples per food à 25 g) cannot be excluded in our dataset. Only one representative of indistinguishable isolates per sampled food should be used for analyses of association and clusters. This requires exact labeling of each sample per food and food-associated surface.

## Conclusion

In conclusion, routine WGS of obligatorily provided isolates of *L. monocytogenes* showed to be useful in promptly generating hypotheses on the sources of invasive listeriosis. In 2019, ECDC-EFSA recommended to introduce routine WGS of human and non-human *L. monocytogenes* isolates across Europe (European Centre for Disease Prevention and Control [ECDC] et al., 2019) to generate a European-wide molecular typing database (Nielsen et al., 2017). Currently, in the year 2019, Austria is one of the three EU countries having already established this routine WGS of all isolates for surveillance of *L. monocytogenes* in 2017 (European Centre for Disease Prevention and Control [ECDC] et al., 2019). By making all isolates of *L. monocytogenes* available for typing by law in 2014, Austria is likely to capture the majority of isolates for continuous cluster analysis, whether they originated from food or human sources, and from private or public laboratories. Austria can expect by using its surveillance system in the upcoming years to detect more clusters, to identify unrecognized sources of *L. monocytogenes*, to link more cases of listeriosis with a likely source and to stop outbreaks earlier, as it has been observed by the Centers for Disease Control and Prevention (CDC) following implementation of a real-time WGS-based surveillance of *L. monocytogenes* in 2013 (Jackson et al., 2016).

## Data Availability Statement

The datasets generated for this study are available on request to the corresponding author.

## Ethics Statement

Ethical review and approval was not required for the study on human participants in accordance with the local legislation and institutional requirements. Written informed consent for participation was not required for this study in accordance with the national legislation and the institutional requirements.

## Author Contributions

WR, AP, DS, and AC designed the study. DS, SH, AC, and FA performed the epidemiological study. AC, AP, and WR performed the WGS analysis. AC and DS analyzed the data. AC, DS, WR, AP, FA, and SH wrote the manuscript. All authors read and approved the final manuscript.

## Conflict of Interest

The authors declare that the research was conducted in the absence of any commercial or financial relationships that could be construed as a potential conflict of interest.

## Footnotes

1 https://bigsdb.pasteur.fr/listeria/

## References

[B1] Buchanan R. L. Gorris L. G. M. Hayman M. M. Jackson T. C. Whiting R. C. (2017). A review of *Listeria monocytogenes*: an update on outbreaks, virulence, dose-response, ecology, and risk assessments. *Food Control* 75 1–13. 10.1016/j.foodcont.2016.12.016

[B2] Chaitiemwong N. Hazeleger W. C. Beumer R. R. Zwietering M. H. (2014). Quantification of transfer of *Listeria monocytogenes* between cooked ham and slicing machine surfaces. *Food Control* 44 177–184. 10.1016/j.foodcont.2014.03.056

[B3] Chenal-Francisque V. Lopez J. Cantinelli T. Caro V. Tran C. Leclercq A. (2011). Worldwide distribution of major clones of *Listeria monocytogenes*. *Emerg. Infect. Dis.* 17 1110–1112. 10.3201/eid/1706.101778 21749783 PMC3358213

[B4] De Cesare A. Parisi A. Mioni R. Comin D. Lucchi A. Manfreda G. (2017). *Listeria monocytogenes* circulating in rabbit meat products and slaughterhouses in Italy: prevalence data and comparison among typing results. *Foodborne Pathog. Dis.* 14 167–176. 10.1089/fpd.2016.2211 28067541

[B5] de Noordhout C. M. Devleesschauwer B. Angulo F. J. Verbeke G. Haagsma J. Kirk M. (2014). The global burden of listeriosis: a systematic review and meta-analysis. *Lancet Infect. Dis.* 14 1073–1082. 10.1016/S1473-3099(14)70870-9 25241232 PMC4369580

[B6] Doumith M. Buchrieser C. Glaser P. Jacquet C. Martin P. (2004). Differentiation of the major *Listeria monocytogenes* serovars by multiplex PCR. *J. Clin. Microbiol.* 42 3819–3822. 10.1128/JCM.42.8.3819-3822.2004 15297538 PMC497638

[B7] Dreyer M. Aguilar-Bultet L. Rupp S. Guldimann C. Stephan R. Schock A. (2016). *Listeria monocytogenes* sequence type 1 is predominant in ruminant rhombencephalitis. *Sci. Rep.* 6:36419. 10.1038/srep36419 27848981 PMC5111077

[B8] Ebner R. Stephan R. Althaus D. Brisse S. Maury M. Tasara T. (2015). Phenotypic and genotypic characteristics of *Listeria monocytogenes* strains isolated during 2011–2014 from different food matrices in Switzerland. *Food Control* 57 321–326. 10.1016/j.foodcont.2015.04.030

[B9] European Centre for Disease Prevention and Control [ECDC], (2016). *Annual Epidemiological Report – Listeriosis, 2016.* Stockholm: European Centre for Disease Prevention and Control.

[B10] European Centre for Disease Prevention and Control [ECDC], European Food Safety Authority [EFSA], Van Walle I. Guerra B. Borges V. Carriço J. A. (2019). EFSA and ECDC technical report on the collection and analysis of whole genome sequencing data from food-borne pathogens and other relevant microorganisms isolated from human, animal, food, feed and food/feed environmental samples in the joint ECDC–EFSA molecular typing database. *EFSA Support. Publ.* 16:1337E 10.2903/sp.efsa.2019.EN-1337

[B11] Fagerlund A. Langsrud S. Moen B. Heir E. Møretrø T. (2018). Complete genome sequences of six *Listeria monocytogenes* sequence type 9 isolates from meat processing plants in Norway. *Genome Announc.* 6 e00016–18. 10.1128/genomeA.00016-18 29449378 PMC5814496

[B12] Gómez D. Iguácel L. P. Rota M. C. Carramiñana J. J. Ariño A. Yangüela J. (2015). Occurrence of *Listeria monocytogenes* in Ready-to-Eat meat products and meat processing plants in Spain. *Foods* 4 271–282. 10.3390/foods4030271 28231204 PMC5224534

[B13] Halbedel S. Prager R. Fuchs S. Trost E. Werner G. Flieger A. (2018). Whole-genome sequencing of recent isolates from Germany reveals population structure and disease clusters. *J. Clin. Microbiol.* 56 e119–18. 10.1128/jcm.00119-18 29643197 PMC5971532

[B14] Henri C. Félix B. Guillier L. Leekitcharoenphon P. Michelon D. Mariet J. F. (2016). Population genetic structure of *Listeria monocytogenes* strains as determined by pulsed-field gel electrophoresis and multilocus sequence typing. *Appl. Environ. Microbiol.* 82 5720–5728. 10.1128/AEM.00583-16 27235443 PMC5007763

[B15] Huang Y. T. Ko W. C. Chan Y. J. Lu J. J. Tsai H. Y. Liao C. H. (2015). Disease burden of invasive listeriosis and molecular characterization of clinical isolates in Taiwan, 2000-2013. *PLoS One* 10:e0141241. 10.1371/journal.pone.0141241 26555445 PMC4640856

[B16] Jackson B. R. Tarr C. Strain E. Jackson K. A. Conrad A. Carleton H. (2016). Implementation of nationwide real-time whole-genome sequencing to enhance listeriosis outbreak detection and investigation. *Clin. Infect. Dis.* 63 380–386. 10.1093/cid/ciw242 27090985 PMC4946012

[B17] Knudsen G. M. Nielsen J. B. Marvig R. L. Ng Y. Worning P. Westh H. (2017). Genome-wide-analyses of Listeria monocytogenes from food-processing plants reveal clonal diversity and date the emergence of persisting sequence types. *Environ. Microbiol. Rep.* 9 428–440. 10.1111/1758-2229.12552 28574206

[B18] Kuch A. Goc A. Belkiewicz K. Filipello V. Ronkiewicz P. Gołȩbiewska A. (2018). Molecular diversity and antimicrobial susceptibility of *Listeria monocytogenes* isolates from invasive infections in Poland (1997–2013). *Sci. Rep.* 8:14562. 10.1038/s41598-018-32574-0 30267005 PMC6162231

[B19] Linke K. Rückerl I. Brugger K. Karpiskova R. Walland J. Muri-Klinger S. (2014). Reservoirs of listeria species in three environmental ecosystems. *Appl. Environ. Microbiol.* 80 5583–5592. 10.1128/AEM.01018-14 25002422 PMC4178586

[B20] Lüth S. Kleta S. Al Dahouk S. (2018). Whole genome sequencing as a typing tool for foodborne pathogens like *Listeria monocytogenes* – The way towards global harmonisation and data exchange. *Trends Food Sci. Technol.* 73 67–75. 10.1016/j.tifs.2018.01.008

[B21] Maury M. M. Bracq-Dieye H. Huang L. Vales G. Lavina M. Thouvenot P. (2019). Hypervirulent *Listeria monocytogenes* clones’ adaption to mammalian gut accounts for their association with dairy products. *Nat. Commun.* 10:2488. 10.1038/s41467-019-10380-0 PMC6554400 31171794

[B22] Meier A. B. Guldimann C. Markkula A. Pöntinen A. Korkeala H. Tasara T. (2017). Comparative phenotypic and genotypic analysis of Swiss and Finnish *Listeria monocytogenes* isolates with respect to benzalkonium chloride resistance. *Front. Microbiol.* 8:397. 10.3389/fmicb.2017.00397 28386248 PMC5362634

[B23] Moura A. Tourdjman M. Leclercq A. Hamelin E. Laurent E. Fredriksen N. (2017). Real-time whole-genome sequencing for surveillance of *Listeria monocytogenes*, France. *Emerg. Infect. Dis.* 23 1462–1470. 10.3201/eid2309.170336 28643628 PMC5572858

[B24] Nastasijevic I. Milanov D. Velebit B. Djordjevic V. Swift C. Painset A. (2017). Tracking of *Listeria monocytogenes* in meat establishment using Whole Genome Sequencing as a food safety management tool: a proof of concept. *Int. J. Food Microbiol.* 257 157–164. 10.1016/j.ijfoodmicro.2017.06.015 28666130

[B25] Nielsen E. M. Björkman J. T. Kiil K. Grant K. Dallman T. Painset A. (2017). Closing gaps for performing a risk assessment on *Listeria monocytogenes* in ready-to-eat (RTE) foods: activity 3, the comparison of isolates from different compartments along the food chain, and from humans using whole genome sequencing (WGS) analysis. *EFSA Support. Publ.* 14:1151E 10.2903/sp.efsa.2017.EN-1151

[B26] Nwaiwu O. Moura A. Thouvenot P. Rees C. Leclercq A. Lecuit M. (2017). Draft Genome sequences of *Listeria monocytogenes*, isolated from fresh leaf vegetables in Owerri city, Nigeria. *Genome Announc.* 5 e354–17. 10.1128/genomeA.00354-17 28572306 PMC5454189

[B27] Pietzka A. Allerberger F. Murer A. Lennkh A. Stöger A. Cabal Rosel A. (2019). Whole genome sequencing based surveillance of *L. monocytogenes* for early detection and investigations of listeriosis outbreaks. *Front. Public Health* 7:139. 10.3389/fpubh.2019.00139 31214559 PMC6557975

[B28] Pontello M. Gori M. Ciceri G. Amato E. (2016). Letter to the editor of infection in response to de Francesco et al., a cluster of invasive listeriosis in Brescia, Italy. *Infection* 44 819–821. 10.1007/s15010-016-0931-x 27506566

[B29] Radoshevich L. Cossart P. (2017). *Listeria monocytogenes*: towards a complete picture of its physiology and pathogenesis. *Nat. Rev. Microbiol.* 16 32–46. 10.1038/nrmicro.2017.126 29176582

[B30] Ragon M. Wirth T. Hollandt F. Lavenir R. Lecuit M. Le Monnier A. (2008). A new perspective on *Listeria monocytogenes* evolution. *PLoS Pathog.* 4:e1000146. 10.1371/journal.ppat.1000146 18773117 PMC2518857

[B31] Ruppitsch W. Pietzka A. Prior K. Bletz S. Fernandez H. L. Allerberger F. (2015). Defining and evaluating a core genome multilocus sequence typing scheme for whole-genome sequence-based typing of *Listeria monocytogenes*. *J. Clin. Microbiol.* 53 2869–2876. 10.1128/jcm.01193-15 26135865 PMC4540939

[B32] Salcedo C. Arreaza L. Alcala B. de la Fuente L. Vazquez J. A. (2003). Development of a multilocus sequence typing method for analysis of *Listeria monocytogenes* clones. *J. Clin. Microbiol.* 41 757–762. 10.1128/jcm.41.2.757-762.2003 12574278 PMC149676

[B33] Schmid D. Allerberger F. Huhulescu S. Pietzka A. Amar C. Kleta S. (2014). Whole genome sequencing as a tool to investigate a cluster of seven cases of listeriosis in Austria and Germany, 2011-2013. *Clin. Microbiol. Infect.* 20 431–436. 10.1111/1469-0691.12638 24698214 PMC4232032

[B34] Sosnowski M. Lachtara B. Wieczorek K. Osek J. (2019). Antimicrobial resistance and genotypic characteristics of *Listeria monocytogenes* isolated from food in Poland. *Int. J. Food Microbiol.* 289 1–6. 10.1016/j.ijfoodmicro.2018.08.029 30189331

[B35] Zuber I. Lakicevic B. Pietzka A. Milanov D. Djordjevic V. Karabasil N. (2019). Molecular characterization of *Listeria monocytogenes* isolates from a small-scale meat processor in Montenegro, 2011–2014. *Food Microbiol.* 79 116–122. 10.1016/j.fm.2018.12.005 30621866

