# Supplementary figures and images for: Isolate-Based Surveillance of Listeria monocytogenes by Whole Genome Sequencing in Austria
Source: Front Microbiol. 2019 Oct 1;10:2282. doi: 10.3389/fmicb.2019.02282 (PMC6779813; doi:10.3389/fmicb.2019.02282)

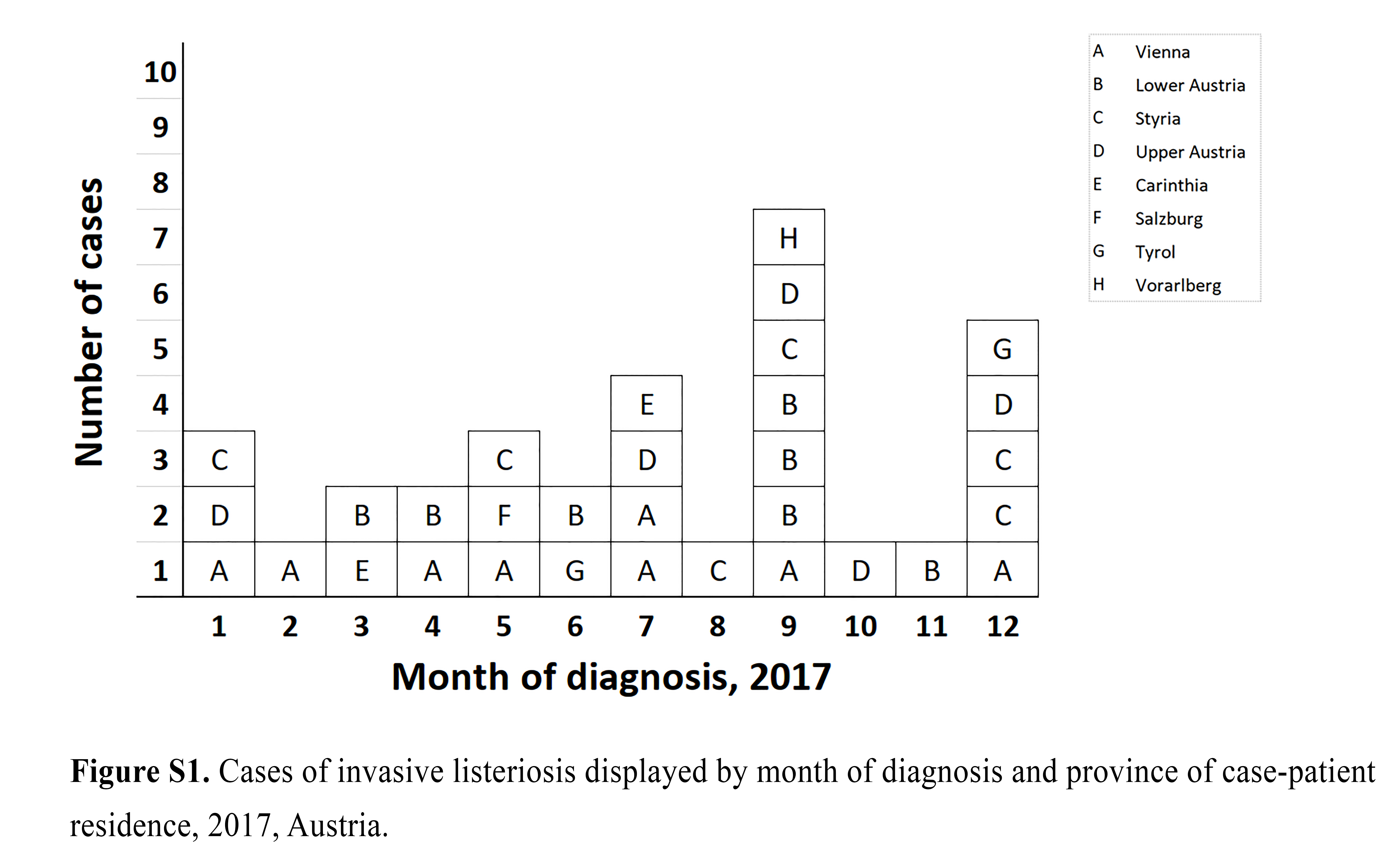

Supplement: Supplementary file 1 [file Image_1.JPEG]
