# Supplementary material for: Isolate-Based Surveillance of Listeria monocytogenes by Whole Genome Sequencing in Austria
Source: Front Microbiol. 2019 Oct 1;10:2282. doi: 10.3389/fmicb.2019.02282 (PMC6779813; doi:10.3389/fmicb.2019.02282)
Supplement: Supplementary file 2 [file Table_1.DOCX]

Supplementary Material

# Supplementary Table

**Table S1.** The ten most prevalent STs among the categories food, food-associated surface and other.

| **ST** | **Food (n=832)** | | **ST** | **Food-associated surfaces (n=849)** | | **ST** | **Other source (n=63)** | |
| --- | --- | --- | --- | --- | --- | --- | --- | --- |
|  | **n** | **%** |  | **n** | **%** |  | **n** | **%** |
| 9 | 109 | 13.1 | 451 | 572 | 67.4 | 1 | 14 | 22.2 |
| 121 | 99 | 11.9 | 8 | 56 | 6.6 | 451 | 5 | 7.9 |
| 511 | 77 | 9.3 | 1462 | 37 | 4.4 | 517 | 5 | 7.9 |
| 8 | 74 | 8.9 | 37 | 24 | 2.8 | 2 | 4 | 6.3 |
| 37 | 60 | 7.2 | 207 | 22 | 2.6 | 5 | 4 | 6.3 |
| 7 | 42 | 5 | 511 | 18 | 2.1 | 26 | 4 | 6.3 |
| 451 | 39 | 4.7 | 21 | 17 | 2 | 4 | 3 | 4.8 |
| 21 | 30 | 3.6 | 3 | 17 | 2 | 7 | 3 | 4.8 |
| 504 | 30 | 3.6 | 20 | 16 | 1.9 | 8 | 3 | 4.8 |
| 155 | 29 | 3,5 | 121 | 9 | 1.1 | 511 | 3 | 4.8 |
